# Genome-wide identification of SSR markers from coding regions for endangered *Argania spinosa* L. skeels and construction of SSR database: AsSSRdb

**DOI:** 10.1093/database/baae118

**Published:** 2024-11-27

**Authors:** Karim Rabeh, Najoua Mghazli, Fatima Gaboun, Abdelkarim Filali-Maltouf, Laila Sbabou, Bouchra Belkadi

**Affiliations:** Oasis Systems Research Unit, Regional Center of Agricultural Research of Errachidia, National Institute of Agricultural Research, Avenue Ennasr, BP 415 Rabat Principale, Rabat 10090, Morocco; Microbiology and Molecular Biology Team, Center of Plant and Microbial Biotechnologies, Biodiversity and Environment, Faculty of Sciences, Mohammed V University, 4 Avenue Ibn Batouta, B.P. 1014, Rabat 10000, Morocco; Microbiology and Molecular Biology Team, Center of Plant and Microbial Biotechnologies, Biodiversity and Environment, Faculty of Sciences, Mohammed V University, 4 Avenue Ibn Batouta, B.P. 1014, Rabat 10000, Morocco; Biotechnology Unit, National Institute for Agronomic Research (INRA), Avenue Ennasr, BP 415 Rabat Principale, Rabat 10000, Morocco; Microbiology and Molecular Biology Team, Center of Plant and Microbial Biotechnologies, Biodiversity and Environment, Faculty of Sciences, Mohammed V University, 4 Avenue Ibn Batouta, B.P. 1014, Rabat 10000, Morocco; Microbiology and Molecular Biology Team, Center of Plant and Microbial Biotechnologies, Biodiversity and Environment, Faculty of Sciences, Mohammed V University, 4 Avenue Ibn Batouta, B.P. 1014, Rabat 10000, Morocco; Microbiology and Molecular Biology Team, Center of Plant and Microbial Biotechnologies, Biodiversity and Environment, Faculty of Sciences, Mohammed V University, 4 Avenue Ibn Batouta, B.P. 1014, Rabat 10000, Morocco

## Abstract

Microsatellites [simple sequence repeats (SSRs)] are one of the most widely used sources of genetic markers, particularly prevalent in plants. Despite their importance in various applications, a comprehensive genome-wide identification of coding sequence (CDS)-associated SSR markers in the *Argania spinosa* L. genome has yet to be conducted. In this study, 66 280 CDSs containing 5351 SSRs within 4535 *A. spinosa* L. CDSs were identified. Among these, tri-nucleotide motifs (58.96%) were the most common, followed by hexa-nucleotide (15.71%) and di-nucleotide motifs (13.32%). The predominant SSR motif in the tri-nucleotide category was AAG (24.4%), while AG (94.1%) was the most abundant among di-nucleotide repeats. Furthermore, the extracted CDSs containing SSRs were subjected to functional annotation; 3396 CDSs (74.88%) exhibited homology with known proteins, 3341 CDSs (73.7%) were assigned Gene Ontology terms, 1004 CDSs were annotated with Enzyme Commission numbers, and 832 (18.3%) were annotated with KEGG pathways. A total of 3475 primer pairs were designed, out of which 3264 were successfully validated *in silico* against the *A. spinosa* L. genome, with 99.6% representing high-resolution markers yielding no more than three products. Additionally, the SSR markers demonstrated a low rate of transferability through *in-silico* verification in two species within the *Sapotaceae* family. Furthermore, we developed an online database, the “*Argania spinosa* L. SSR database: https://as-fmmdb.shinyapps.io/asssrdb/” (AsSSRdb) to provide access to the CDS-associated SSRs identified in this study. Overall, this research provides valuable marker resources for DNA fingerprinting, genetic studies, and molecular breeding in argan and related species.

**Database URL:** https://as-fmmdb.shinyapps.io/asssrdb/

## Introduction

The argan tree (*Argania spinosa* L.), indigenous to Southwestern Morocco and a member of the *Sapotaceae* family, thrives in semi-arid and arid environments. It is of paramount significance to local communities for its botanical, ecological, economic, and social value [1]. The tree is extensively used by the local population for nutritional, medicinal, and cosmetic purposes. The extraction of the valuable argan oil from its seeds, which is enriched with unsaturated fatty acids, is the tree’s main product, with wide applications in food, cosmetics, and medicine [2]. *Argania spinosa* L. is both monoecious and allogamous [3], with a diploid nature and a karyotype of 2n = 24 [4]. Recent studies have shown that this species exhibits chromosomal number variation (2n = 20, 22, 24) with a stable diploid ploidy level (2n = 2x), indicating significant genetic diversity in chromosomal numbers [5].

Various methods have been used to assess the genetic diversity of the argan tree, including morpho-chemical, biochemical, and molecular markers [6–8]. DNA markers have proven to be superior in accuracy and reliability for this purpose, aligning with the broader trend in molecular genetics to address ecological and conservation concerns in various forest species over the past decades [9]. Multiple molecular methods, including amplified fragment length polymorphism, inter-simple sequence repeat, inter-retrotransposon amplified polymorphism, sequence-related amplified polymorphism, retrotransposon-microsatellite amplified polymorphism, and simple sequence repeats (SSRs), have been employed to assess genetic variability in *A. spinosa* L [10–16].

El Mousadik and Petit [17, 18] initiated the examination of genetic diversity in *A. spinosa* L. using isozyme markers and Polymerase Chain Reaction-Restriction Fragment Length Polymorphism of chloroplast DNA, followed by a study employing Random Amplified Polymorphic DNA (RAPD) [10]. Subsequent efforts have focused on developing SSR markers. Majourhat *et al*. [10] generated the first set of twenty SSRs (non-specific) derived from two species of the *Sapotaceae* family. Specifically, twelve primer pairs from *Manilkara huberi* (Ducke) Standl [19] and eight from *Vitellaria paradoxa* C.F. Gaertn [20] were designed for transferability to amplify microsatellite loci across 38 argan trees, yielding polymorphism in only 6 SSRs. A second set of 79 SSRs (specific to *A. spinosa*) from enriched repeat regions of genomic libraries was developed by El Bahloul *et al*. [21] to assess the genetic diversity of 150 argan trees, of which only 11 SSR markers displayed high polymorphism.

Compared to other molecular markers, SSRs or microsatellite markers (one to six base pairs in length) are considered the most reliable due to their ubiquity in the nuclear genome of eukaryotes, including coding and non-coding regions [22, 23]. These SSRs are powerful tools for assessing genetic diversity and are applied in population genetic structure analysis, drought stress investigation, understanding adaptive genetic variation, and ecotype differentiation [11, 15, 24–26].

Genome-wide molecular markers, particularly SSRs, have been extensively developed for numerous sequenced plant genomes, although comprehensive molecular marker databases are available for only a limited number of species. For instance, the Cotton Microsatellite Database catalogs 5484 SSR markers derived from nine major cotton microsatellite projects Similarly, the Kazusa Marker Database provides linkage and physical maps, along with data on approximatively 68 000 SSR primers for 14 agronomically important crops [27]. In addition, the Pigeon Pea Microsatellite Database (PIPEMicroDB) records 123 387 short tandem repeats identified in the pigeon pea genome [28], while the Foxtail Millet Marker Database (FmMDb) catalogs 21 315 genomic SSRs, 447 genic SSRs, and 96 intron length polymorphisms [29]. The Chickpea Microsatellite Database (CicArMiSatDB), developed from chickpea genome data, provides extensive SSR information [30].

The SSRome database is a large-scale resource encompassing 45.1 million microsatellite markers across diverse taxa, including plants, metazoans, archaea, bacteria, viruses, fungi, and protozoa. It covers microsatellites from 6533 organisms across nuclear, mitochondrial, and chloroplast genomes [31]. The Pineapple Genomics Database integrates genomic, transcriptomic, and SSR marker data for pineapple [32], while the Lily Genomic Database provides SSR and SNP markers specific to lily species [33].

TriticeaeSSRdb, a comprehensive and user-friendly platform, contains 3 891 705 SSR markers from 21 species [34]. In addition, the MicroSatellite DataBase (MSDB), a broad database covering over 46 000 genomes, hosts one of the largest collections of microsatellites, including 1 456 366 SSRs from the *Argania spinosa* genome [35]. However, many of these databases present limitations, including: (i) a lack of associated gene function annotations, (ii) missing transferability information, which is critical for selecting high-quality markers for *in silico* applications, and (iii) the absence of data on the specific genomic locations of SSR motifs, particularly in coding regions. In general, existing marker databases provide a limited range of information for each marker, such as forward and reverse primer sequences, SSR types, primer annealing temperatures, and PCR product sizes.

The current study aims to fill these gaps by providing a comprehensive report on SSR marker mining through *in silico* analysis using the draft genome sequence of *Argania spinosa* L. Specifically, the study focuses on identifying SSRs in coding regions and predicting their associated gene functions. Furthermore, it seeks to develop novel SSR markers from coding regions, validate them *in silico*, and assess their cross-species transferability. A user-friendly database of SSRs for *Argania spinosa* has also been developed, providing a valuable resource for future studies on population genetics, genetic fingerprinting, and the regulatory mechanisms governing functional genes in this species.

## Materials and methods

### *In-silico* SSR mining in *A. spinosa* L. genome

The draft genome sequence of the argan tree (*A. spinosa* L.), consisting of 186 325 scaffolds, is available from the National Center for Biotechnology Information (NCBI) under accession number GCA_003260245.2_ASM326024v2. This genome, assembled at the scaffold level (QLOD02000001 to QLOD02186325), was obtained from the NCBI Genomes FTP (FTP File Transfer Protocol) site (ftp://ftp.ncbi.nlm.nih.gov/genomes/).

**Figure 1.**
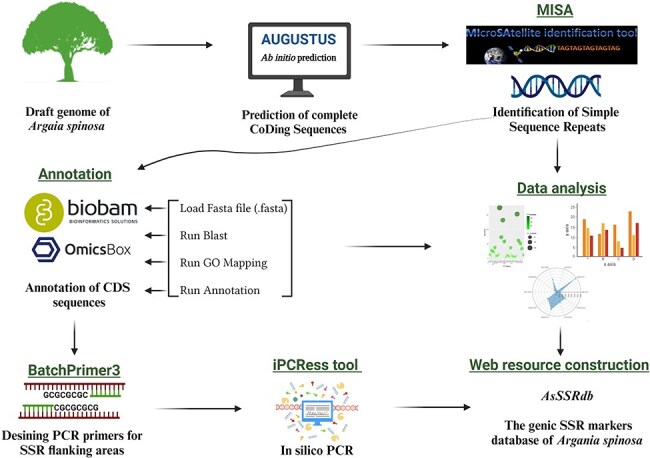
The workflow and bioinformatics pipeline employed in this study to systematically developp genome-wide SSR markers in *A. spinosa* L.

To predict genes within the argan tree genome, an *ab-initio* prediction approach (Fig. 1) was employed using the Augustus web server [36]. Subsequently, the MIcro-SAtellite (MISA) program (http://pgrc.ipk-gatersleben.de/misa/, accessed on 30 March 2023) [37] was used to identify and locate SSR motifs (Fig. 1), including perfect SSRs and compound motifs, within the coding regions (predicted genes). In compound sequences, the distance between two SSRs was less than 100 base pairs (bp). MISA criteria considered microsatellites with basic motifs ranging from one to six nucleotides (bp), with minimum repeat units of ten for mononucleotides (MNN), six for dinucleotides (DIN), and five for tri-(TRN), tetra-(TTN), penta-(PNN), and hexanucleotide (HXN) repeats.

### Functional annotations of genes containing SSR

Functional annotation and gene ontology (GO) analysis were carried out using Omics-Box BioBam software version 3.0 (https://www.biobam.com/omicsbox/) [38]. Annotation was performed using the output file of the *ab initio* gene prediction process. Protein sequences were aligned against the NCBI non-redundant protein sequence through BLASTp search database in the OmicsBox program, employing default settings. The results from the local BLAST search were used for subsequent analyses, including mapping, annotation, GO and Enzyme Commission (EC) analysis (Fig. 1). The GO terms for the annotated sequences were predicted based on the following parameters: cut-off 55, E-value 1 × 10e − 6, GO weight of 5, and HSP-hit coverage cut-off 0. The GO terms were further categorized into three main classes: biological process, cellular component and molecular function using the KEGG database.

### Primer designing and *in-silico* PCR

PCR primers were designed based on the flanking region of the SSR using Batch Primer 3 v1.0.

(https://probes.pw.usda.gov/batchprimer3/index.html/, accessed on 1 November 2023) (Fig. 1). One pair of primers was designed for each SSR repeat. Primer design constraints included: (i) primer length ranging from 19 to 27 bp, with 20 bp being optimal; (ii) annealing temperature ranging from 55°C to 65°C, with 60°C being optimal; (iii) PCR amplification products varying from 100 bp to 400 bp; and (iv) GC content spanning 40% to 60%, with 50% as optimal. All other parameters were set to the default values of the program. The designed primers were subjected to *in-silico* PCR against the *A. spinosa* L. genome sequence using the iPCRess (In-silico PCR Experiment Simulation System) tool from the Exonerate v2.2.0 software package [39] to generate the amplicons. The electronic PCR parameters were 0 bp mismatch, 0 bp gap, and 0–5000 bp product size. Furthermore, the designed primers were evaluated for *in-silico* amplification in species of the *Sapotaceae* family using the iPCRess tool to assess their transferability.

### AsSSRdb, an SSR web-genic resource for *A. spinosa* L.

In this study, a comprehensive and user-friendly web application was developed using the Shiny package (version 1.8.0) in R (version 4.3.2) to facilitate the search and analysis of functional molecular markers obtained from *A. spinosa* L. The application features a “Search by Parameters” tab, allowing users to filter data using specific criteria, thereby providing a streamlined approach to data retrieval. Additionally, the “Advanced Search” tab offers more intricate filtering options, enabling users to refine their search based on additional parameters. This web application is available at https://as-fmmdb.shinyapps.io/asssrdb/.

### Data analysis

Extensive graphical representations were created using both R (v1.3.1093) programming and SRplot. In R, our approach involved data preparation and the use of the ggplot2 (v3.4.4) package, known for its flexibility in data visualization. Further visualizations were generated through https://www.bioinformatics.com.cnv (last accessed on 5 January 2024), an online platform dedicated to data analysis and visualization [40].

## Results

**Figure 2.**
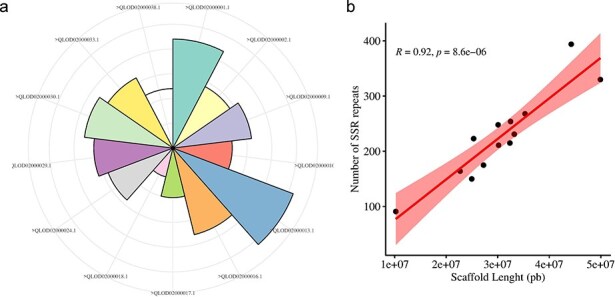
Distribution and number of SSRs on *A. spinosa* scaffolds (a), and their correlation with schaffold lengths.

**Figure 3.**
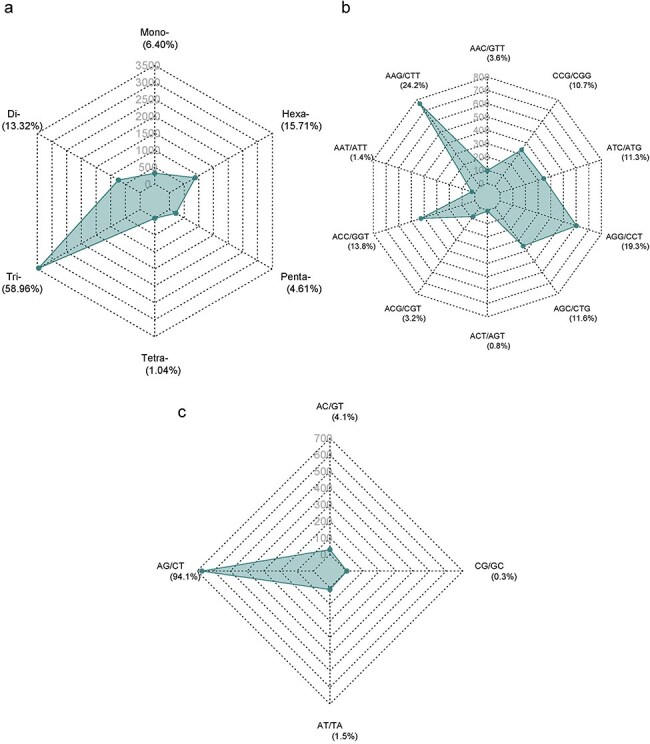
Distribution of SSR numbers in *A. spinosa* L. predicted genes: (a) all types of SSR motifs; (b and c) predominant repeat motifs.

The Augustus predicted a total of 66 280 coding sequences (CDSs) in our study, which were identified within the 715.1 Mb genome of *A. spinosa* L. The identified CDS collectively spanned a total length of 59 704 245 bp, constituting 11.98% of the entire genome. From 4535 CDS, 5351 microsatellite repeats (perfect and compound) were identified and distributed among 13 *A. spinosa* L. scaffolds out of a total of 1015 scaffolds. Of these, 657 sequences contained more than one SSR, and 395 were found in compound formation. The SSR blocks exhibited a typical length of 19.45 bp, with a relative abundance and density of 89.9 loci/Mb and 1749.6 bp/Mb, respectively (Table 1). Upon analyzing the distribution in the 13 scaffolds, the largest and smallest numbers of SSR types were mapped to scaffolds QLOD02000013.1 (394) and QLOD02000018.1 (94), respectively (Fig. 2a). The remaining scaffolds, excluding the 13 mentioned above, had SSR types ranging from 1 to 6 (Supplementary Table S1). Additionally, Pearson correlation analysis revealed a significant positive association between scaffold length and the number of SSRs in each scaffold (*r* = 0.92, *P* < .01) (Fig. 2b). Regarding the number of SSR motifs, TRN were the most abundant (3155, 58.96%), followed by HXN (841, 15.71%), DIN (713, 13.32%), MNN (339, 6.40%), PNN (247, 4.61%), and TTN (56, 1.04%) (Fig. 3a). Relative abundance and density were determined for each repeat type, with TRN exhibiting the greatest occurrence frequency and density of 53.0 loci/Mb and 780.5 bp/Mb, respectively, followed by HXN (14.1 loci/Mb), DIN (12.0 loci/Mb), MNN (5.7 loci/Mb), PNN (4.2 loci/Mb), and TTN showing the lowest prevalence of 1.0 loci/Mb and a density of 11.3 bp/Mb (Table 2).

**Table 1.** Statistics of microsatellites mined from coding regions of *A. spinosa* L.

SSR mining	Description	Number
Total number of coding examined	Counts	66 280
Total size of examined sequences	A + T + C + G + N (bp)	59 704 245
Total valid length of sequences	A + T + C + G (bp)	59 487 565
Unkown bases (Ns) in sequences	Bp	216 680
Percentage of unkown bases	Percentage (%)	0.36
GC content	(G + C)/(A + T + C + G) not include Ns (%)	45.35
Number total of SSRs	Counts	4535
Number of genes with SSRs	Counts	5351
Number of genes with more than 1 SSRs	Counts	657
Number of SSRs present in perfect formation	Counts	4694
Number of SSRs present in compound formation	Counts	395
The average length of SSRs	Total SSR length/total ssr counts (bp)	19.45
The percentage of sequence covered by SSRs	Total SSR length/total sequence length (%)	0.17
Relative abundance	Loci/Mb	89.9
Relative density	pb/Mb	1749.6

**Table 2.** Overall length, abundance, and density of six different types of perfect SSRs in coding regions (predicted genes) and the number of markers designed

SSR Loci				
Motif	Length (pb)	Relative abundance (loci/Mb)	Relative Density (bp/Mb)	Number of designed marker
Mono-	3868	5.7	65.0	0
Di-	9972	12.0	167.6	587
Tri-	46 431	53.0	780.5	2840
Tetra-	672	1.0	11.3	8
Penta-	3715	4.2	62.4	6
Hexa-	14 310	14.1	240.5	34

**Figure 4.**
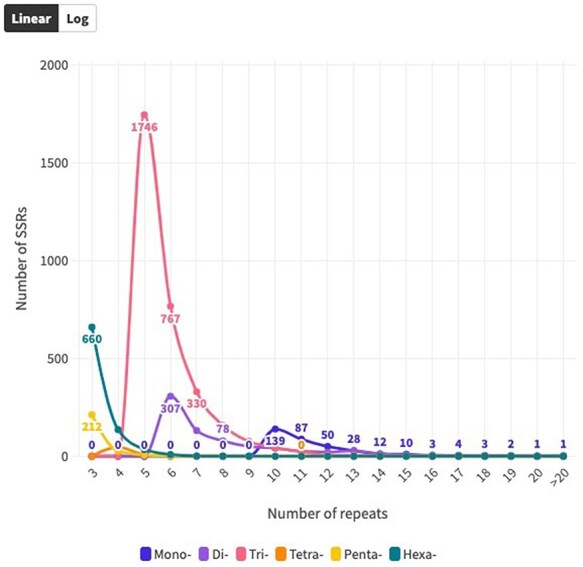
Distribution of SSR repeat number for each SSR type over coding sequences.

The distribution of SSR motifs exhibited notable variation. AAG/CTT was the most common type of TRN repeat, accounting for 24.24% (765 of all TRN repeats). AGG/CCT accounted for 19.27% (608 repeats), while the rare type ACT/AGT accounted for only 0.82% (26 repeats). Among HXN, ACCTCT/AGAGGT was the most common (48, 5.70% of all HXN repeats), followed by ACCGCC/CGGTGG (41 repeats, 4.87%). For DIN, AG/CT (94.1%, 713 of all DIN repeats) was the most common, and CG/CG was the least common with 0.28% (2 repeats). The most abundant PNN and TTN repeats were AAACC/GGTTT (8.5%, 21 repeats) and AGGG/CCCT (25%, 14 repeats), respectively (Fig. 3b and c; Supplementary Table S2). Copy number distribution also varied by repeat type. MNN repeats were mostly concentrated between 10 and more than 20 occurrences, with 10 repeats accounting for 41.0% (139). DIN repeats varied from 6 to 19 occurrences and constituted 91.37% (191 786) of the total DIN SSRs. Among them, 6 repeat types were the most prevalent, accounting for 43.0% (307). TRN repeats were mainly concentrated in 5–15 occurrences, making up 92.67% (38 444) of the total number, with 5 repeats being the most common at 55.34% (1746). TTN repeats predominantly occurred 4–7 times, constituting 90.70% (25 798), with 4 repeats being the most abundant at 83.9% (47). PNN repeats were concentrated in 3–6 occurrences, constituting 95.54% (28 054) of all PNN SSRs, with 3 repeats accounting for 85.8% (212). HXN repeats dominated in the 3–6 occurrences category, constituting 98.90% (9301) of total HXN SSRs, with the three repeats category being the largest at 87.47% (660) (Supplementary Table S1, Fig. 4).

### Functional annotation of genes harboring SSRs

**Figure 5.**
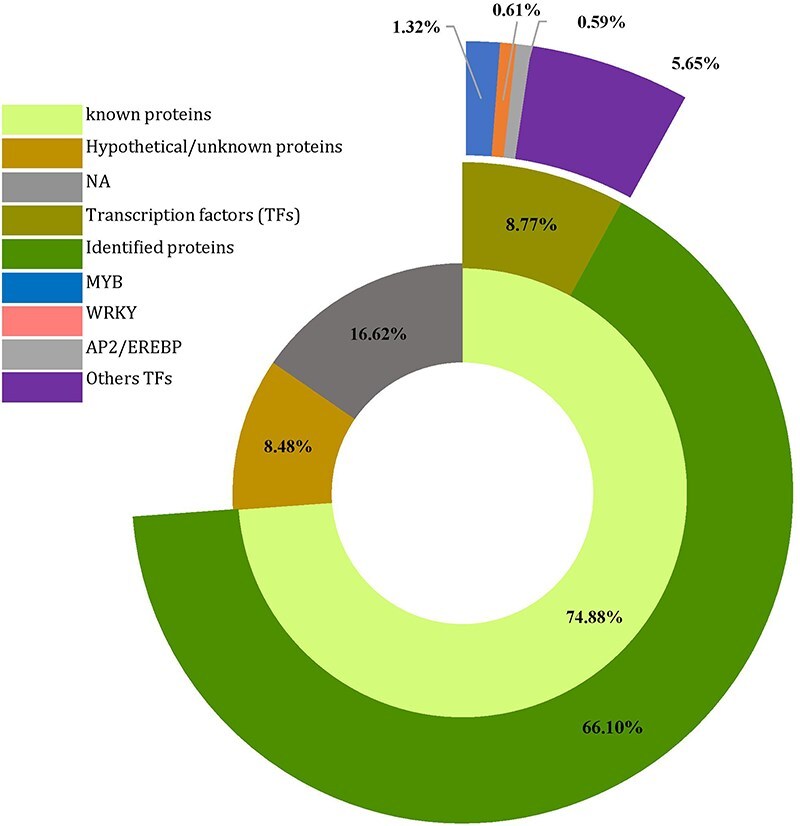
Number of predicted genes and transcription factors harboring SSR in *Agania spinosa* L.

To explore the potential biological processes controlled by the 4535 CDS (predicted genes) housing SSRs (Supplementary Table S3), a homology search was conducted for all SSR-containing genes against proteins in the NCBI database using the Blast tool implemented in OmicsBox. Of these, 3396 CDSs (74.88%) displayed homology to known proteins, whereas 385 (8.48%) showed homology to expressed, hypothetical, or unknown proteins. The remaining 754 sequences (16.62%) had no homology to any known proteins (Fig. 5, Supplementary Table S3). Additionally, of the 3396 annotated sequences, 398 were associated with transcription regulator activity, representing various families. Among these, the Myeloblastosis (*MYB*) family displayed the highest abundance (60), followed by the *WRKY* (28) and Apetala2/Ethylene Response Factor (*AP2/EREBP*) (27) families, suggesting potential involvement in the regulation of development and metabolism (Fig. 5).

**Figure 6.**
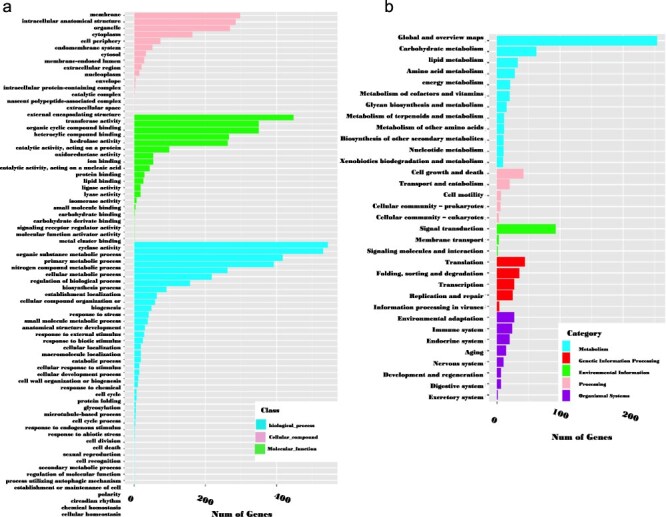
GO classification (a) and KEGG metabolic pathway mapping (b) of 3341 genes containing SSRs.

These genes were annotated using OmicsBox software. According to the GO annotation, 73.7% (3341 out of 4535) of the sequences could be assigned GO numbers. A gene product may be associated with or situated in one or more cellular components, actively involved in one or more biological processes, and perform one or more molecular functions. Consequently, some predicted genes were annotated simultaneously with the three categories. Figure 6a illustrates the classification of GO terms A total of 8110 GO terms were categorized into biological process (2524 GO terms; 38 categories), molecular function (4027 GO terms; 20 categories), and cellular component (1559 GO terms; 16 categories). Based on GO terms, the maximum number of CDSs were present in “transferase activity” and “organic cyclic binding” in the molecular function category; in “organic substance metabolic process” and “primary metabolic process” in the biological process category; and in “membrane” and “intercellular anatomical structure” in the cellular component category (Fig. 6a).

**Figure 7.**
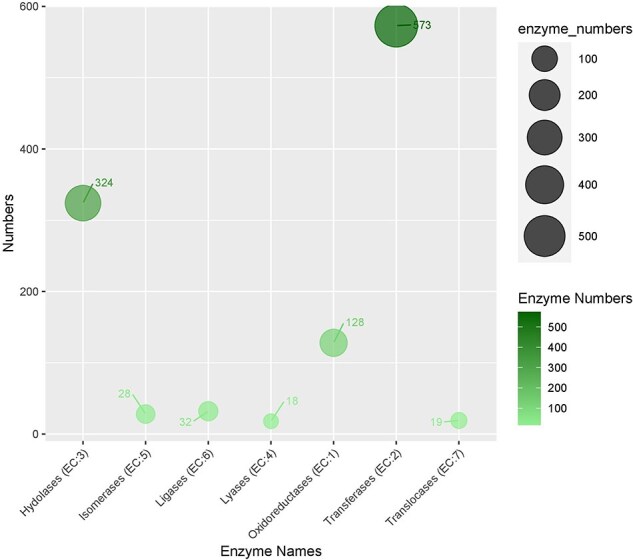
The distribution of enzymes categories in genic sequences harboring SRR.

In the KEGG pathway database, a total of 832 (18.3%) CDSs containing SSR were annotated to 33 KEGG pathways, which were further classified into five major metabolic pathways [metabolism, environmental information processing (EIP), cell process (CP), genetic information processing (GIP), and organismal systems (OS)]. Out of the five categories, more than 53.77% were attributed to the metabolism pathway, mainly carbohydrate metabolism (62), lipid metabolism (33), and amino acid metabolism (28). Additionally, cell growth and death, signal transduction, translation, and environmental adaptation were the most common in the CP, EIP, GIP, and OS categories, respectively (Fig. 6b). Enzyme codes (1122) and enzyme names were assigned to 1004 CDSs. The largest number of CDS encoded transferases (573 enzymes), followed by hydrolases (324), and oxidoreductases (128) (Fig. 7).

### Development SSR, primer *in-silico* PCR and transferability

For each SSR, forward and reverse primer pairs were designed based on the flanking sequences. A total of 3475 SSR markers (SSR loci) were successfully developed in *A. spinosa* L., which account for 69.73% of all identified SSRs (Supplementary Table S4). Of the 3475 SSR markers developed, a large proportion of primer pairs were on TRN (71.37%, 2840) and DIN (16.89%, 587), while HXN (34), TT- (8), and PNN (6) repeats together accounted for less than 1% of the total primer pairs (Table 2).

**Figure 8.**
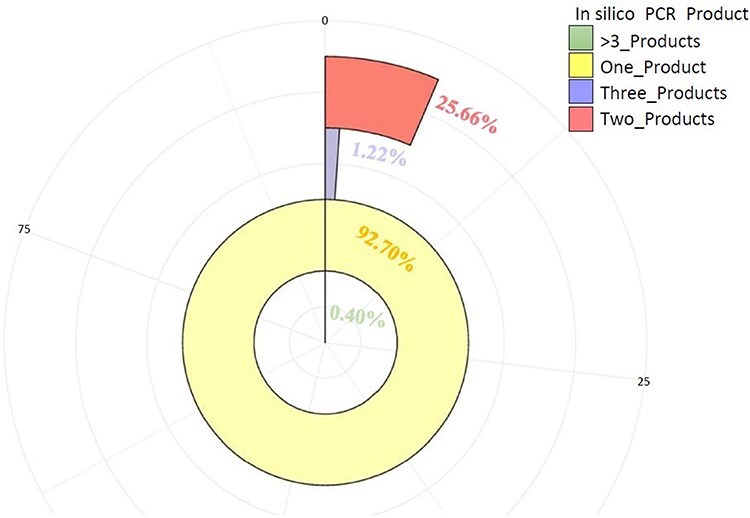
The number of primer pairs and their PCR products (one, two, three, >3) generated *in-silico* against *A. spinosa* L. genome.

These markers (3475) were further tested by *in-silico* PCR using the iPCRess tool against the A. spinosa L. genome to confirm their efficacy. Using the A. spinosa L. genome sequence, 3264 (93.90%) SSR primer pairs successfully identified 3584 sequences that were complementary, with an average of 1.099 alleles per primer pair. Among the total SSR markers (3264), 92.70% (3026) were high-resolution markers in the argan genome, generating one *in-silico* product. Additionally, 5.64% (184) and 1.26% (41) of the SSR markers produced two and three products, respectively, while only 0.40% of the markers (13) generated more than three *in-silico* products (Fig. 8, Supplementary Table S5). Subsequently, these markers (3264) were further assessed for cross-species transferability in two species of the *Sapotaceae* family: *Vitellaria paradoxa* (ASM1991606v1) and *Pouteria glomerata* (ASM3559065v1). As detailed in Table 3, 171 (5.24%) and 142 (4.35%) of the SSRs exhibited transferability to *V. paradoxa* and *P. glomerata*, respectively. Notably, 54 markers were shared between the two species (Supplementary Table S5).

**Table 3.** *In silico* transferability of *A*. *spinosa* L. SSR markers in *Sapotaceae* family

	As a positive control	Related species	
Items	As sequence	Pg sequence	Vp sequence
No of tested primers	3475	3475	3475
No of primers found hits in genome	3263	142	171
Transferability (%)	-	4.35%	5.24%
No of hit sequences in genome	3586	147	187
Genome size (Mb)	690.5	722	667.2
Assembly level	Scaffold	Scaffold	Chromosome
Number of scaffolds/chromosomes	186 325	189 300	109

As: *Argania spinosa* L., Pg: *Pouteria glomerata*, Vp: *Vitellaria paradoxa*.

### AsSSRdb: *Argania spinosa* L. SSR web-genic resource

**Figure 9.**
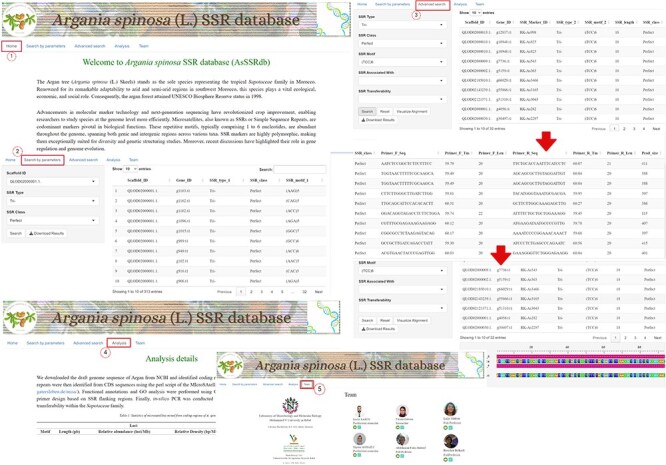
The interface and searching of the *Argan spinosa* L. SSR database “AsSSRdb’.

The *A. spinosa* L. SSR database (AsSSRdb) is a publicly accessible and searchable resource that includes the microsatellite data reported in this study. This database provides convenient access to all genic (CDS regions) SSRs identified in the argan genome. Users can retrieve SSRs through simple searches, such as by SSR type (perfect vs. imperfect) and scaffold ID, or through more advanced searches based on motif type (MNN to HXN), specific motif sequences, or repeat numbers. Search results include detailed information on SSR repeats, including ID, type, motif, length, class, and position, as well as visualization of flanking primers with their characteristics (sequence, Tm, length, and product size). All results can be downloaded in text file format. A detailed workflow for exploring the AsSSRdb and its search features is outlined in Fig. 9.

## Discussion

Molecular markers are powerful genomics tools for exploring genetic variation and identifying genomic regions or genes associated with specific traits in plants [41]. The advent of next-generation sequencing technology has facilitated the completion of whole-genome sequencing for numerous species, providing an unprecedented amount of data for developing a substantial number of SSR molecular markers. Furthermore, the availability of the argan draft genome sequence has significantly enhanced the potential for genome-wide marker development, paving the way for SSR markers spanning the entire genome. The discovery and mining of SSR markers have been successful in various plant species, including *Triticum* spp. [42], *Diospyros oleifera* [43], *Matthiola incana* [44], *Shorea robusta* [45], *Juglans regia* [46, 47], *Beta vulgaris* [48], *Camellia sinensis* L [49, 50]., *Bunium persicum* [51], *Lagenaria siceraria* [52], *Monopterus albus* [53], *Vigna radiata* [41], *Trillium govanianum* [54], banana [55], *Punica granatum* [56], *Cicer arietinum* [57], *Arachis hypogaea* [58], *Asparagus officinalis* [59] *Ziziphus jujuba* [60], *Citrus sinensis* [61], *Clementine mandarin* [62], *Ricinus communis* [63], and *Malus x domestica* Borkh [64] among others. To support data sharing, user-friendly and accessible databases have been developed, including LegumeSSRdb [65], SSRome [31], citSATdb [66], PSSRD [67], TriticeaeSSRdb [34], Kazusa Marker Database [27], and MSDB [35]. In *A. spinosa*, the MSDB is the primary database, containing a large number of SSR markers (1 456 366 SSR) derived from its genome [35]. However, this database has notable limitations, such as primer redundancy, limited transferability, and the absence of gene function information associated with SSRs.

Determining the position of microsatellites within genes (coding regions) is crucial for utilizing transcript SSRs in microsatellite evolution studies and marker development [62]. In this study, we examined 66 280 predicted CDSs for SSR marker identification and development. The high number of CDSs, accounting for 11.98% reflects a significant gene density and suggests that the genome is highly optimized for functional adaptability. This elevated gene content may be attributed to several factors, including the species’ adaptation to arid environments, which could necessitate a large repertoire of genes to cope with various stress conditions. Consequently, the abundant presence of SSRs within these CDSs provides a valuable resource for developing robust molecular markers that can facilitate genetic diversity studies and support breeding programs aimed at enhancing stress resilience. As a result, a total of 5351 SSRs were identified across 13 scaffolds, encompassing 4535 CDSs. Approximately 6.84% of argan CDSs were found to carry SSRs. Notably, the density of SSRs in coding regions (89.9 loci/Mb) exceeded that reported in other species, such as *D. oleifera* (13.48 loci/Mb) [43]. This discrepancy may be due to differences in the size of the sequence datasets analyzed, the mining tools employed, and the parameters used for SSR identification. Additionally, the frequency of SSRs exhibits broad variability among plant species [68].

Among different genic regions, our data revealed that the CDS region had the most dominant repeat types in TRN (58.96%, 3155 SSRs), a trend consistent with findings in other plant species, including *S. robusta* (68.23%, 1939 SSRs), *Stevia rebaudiana* (44.26%, 8789 SSRs), *Anacardium occidentale* (49.12%, 4271 SSRs), and citrus (36%, 8989 SSRs) [45, 62, 69, 70], as well as other species [54, 71–73].

The sequence AAG/CTT, encoding lysine, was found to be the most abundant TRN genic SSR motif type, while ACT/AGT (encoding threonine) repeats were the least abundant (0.82%). The prevalence of AAG/CTT repeat motifs, particularly in coding regions (24.24%), mirrors previous findings in dicots such as *Citrus* [62], *D. oleifera* [43], and *poppy anemone* [74]. Conversely, other studies report that CCG/GGC TRN repeats, encoding proline, dominate in monocots like wheat [73] and *T. govarianum* [54]. As noted by Metzgar *et al*. [75], the prevalence of TRN in CDS regions underscores their evolutionary role in protecting genes from frameshift mutations. Similarly, the most common DIN in the genic region was AG/CT (94.1%). The prevalence of AG/CT motifs in gene sequences has been extensively documented across various species, including citrus, persimmon, eggplant, and mung bean [43, 62, 76, 77]. Genic SSRs, which are present in transcripts, are recognized as significant categories of “functional markers,” derived from functionally characterized sequence motifs [78]. They play a pivotal role in regulating gene expression in both prokaryotic and eukaryotic organisms [79]. Furthermore, genic microsatellite markers have been shown to have higher transferability between related species, making them suitable as anchor markers in comparative genetics [80].

Moreover, we investigated the potential functions of the developed CDS-associated SSR markers through a homology comparison of the genes harboring these SSRs. This analysis revealed that these genes are involved in a range of functions, suggesting that the CDS-associated SSRs may be linked to important biological traits. Specifically, 73.7%, 22.1%, and 18.3% of genes in *A. spinosa* L. were annotated with 78 GO subcategories, 7 enzyme categories, and 33 different classifications in the KEGG database, respectively. The high proportion of GO terms could serve as signature markers and be useful for trait-associated marker selection in argan.

In terms of molecular function, the majority of GOs were associated with “transferase activity; GO:0016740” and “organic cyclic binding; GO:0097159,” indicating their significant roles in various physiological and biochemical processes. Additionally, in the EC categories, most of the developed SSR loci were linked to genes involved in transferases and hydrolases. Previous studies have shown that these enzymes exhibit diverse biochemical activities and undergo specific chemical adaptations in response to various biotic and abiotic stresses [81]. Furthermore, the SSR-associated genes were enriched in “metabolic processes” and ‘organismal systems” pathways in the KEGG database. These findings suggest that the developed gene-associated SSRs are crucial for maintaining genetic diversity and hold promise for future functional studies.

Of the 5351 SSR loci identified, we designed 3475 (64.94%) primers, which is higher than the 1091 primers reported for *Vicia faba* by Khalifa *et al*. [82], 1000 reported in Eggplant (*Solanum melongena*) by Portis *et al*. [77], 150 reported in *Anemone coronaria* [74], and 184 reported in *Medicago truncatula* [83]. However, it is lower than the 3795 primers reported in *Coriandrum sativum* by Tulsani *et al*. [84], the 71 184 reported in flax, *Linum usitatissimum* by Pan *et al*. [85], and the 3607 reported in Lilium by Biswas *et al*. [61]. The designed SSR primers were validated by *in-silico* PCR to assess amplification efficiency. Although validating each primer pair through a thermocycler can be challenging, *in-silico* PCR analysis proves to be a highly valuable method for assessing primer performance and providing additional information regarding specificity [86]. Impressively, a substantial percentage of the designed primers (93.92%, 3264) successfully produced amplicons, while the remaining markers did not. The presence of non-validated markers may be attributed to large introns between primer sites. Despite this, the majority of SSR primer pairs (92.70%) produced a single allele, indicating successful amplification and specificity. However, a small proportion of SSR primers (7.30%) yielded multiple bands. As reported by Khalifa *et al*. (2021), SSR primers yielding a high number of PCR bands may be used for cultivar fingerprinting and differentiating closely related accessions.

To assess the potential transferability and effectiveness of the SSR primers, we conducted *in-silico* amplification for all developed primer pairs. Given that *A. spinosa* L. is the only species within the argan genus, our investigation focused on species from the *Sapotaceae* family, including *V. paradoxa* and *P. glomerata*. In species where genomic resources are limited, the transferability of markers is recognized as a cost-effective approach for developing genetic markers suited to their particular requirements [87, 88]. In our study, approximately 5.24% (171) and 4.35% (142) of the SSR primer pairs demonstrated *in-silico* cross-species amplification in *V. p* and *P. glomerata*, respectively. This rate is relatively low compared to the levels of transferability observed in other species, such as 97% in *Vigna radiata* [76], 94.4–98.8% in *Mangifera indica* [89], and 97.1% in *Pistacia vera* [90]. The modest rate of transferability of genic SSRs can be attributed to two factors. First, it may be linked to the distant phylogenetic/taxonomic relationships of the species (*A. spinosa, V. paradoxa*, and *P. glomerata*). This observation is consistent with the general trend of reduced amplification as evolutionary distances between species increase [91]. Similarly, previous research has shown that SSR markers exhibit high potential for cross-species amplification/transferability in related species within the same genus [63]. Secondly, it could be due to the fact that SSRs are derived from specific regions, the coding parts of the genome. This observation aligns with Scott *et al*. [92], who concluded that genic SSRs generally exhibit higher conservation compared to SSRs from non-genic regions. Conversely, other studies have reported that the highest rates of transferability of genic SSRs are attributed to the higher conservation of genic sequences compared to sequences from anonymous regions of the genome [70, 87, 93].

With the increasing availability of genome-wide microsatellites/SSRs data, there is a crucial need for the development of user-friendly web tools to facilitate easy access and efficient utilization of mined SSRs in genetic studies and crop enhancement. Various databases housing genome-wide SSR information have been established for different plant species [66, 74, 77, 94, 95]. However, until now, genome-wide SSR data for argan have not been accessible. In this study, the genome-wide SSR information generated was integrated into AsSSRdb (**A**rgania **s**pinosa **SSR d**ata**b**ase), enabling the extraction of information specifically related to genic SSRs. This web resource serves a dual purpose: facilitating the selection of microsatellites of a particular type and assisting in the identification of all putative microsatellites associated with functional gene sequences. As such, AsSSRdb stands as the first comprehensive microsatellite database for argan and provides invaluable support to researchers in the argan community, particularly breeders. It provides a platform for the development of novel markers from *in-silico* generated SSRs and the direct use of experimentally validated markers in research programs.

## Conclusion

This study represents the first in-depth characterization of genome-wide SSRs across coding regions for the *A. spinosa* L. draft genome, accompanied by the development of a user-friendly database.

We identified a total of 66 280 CDS, of which 4535 contained SSR motifs. Tri-nucleotide repeats were found to be the most prevalent SSR type, accounting for 58.96% of the identified SSRs. Furthermore, functional annotation was successfully performed on the 4535 annotated CDS, with 3396 (NR, 74.88%), 3341 (GO, 73.7%), 1004 (EC, 22.1%), and 832 (KEGG, 18.3%) CDS annotated through this process.

Out of the 3475 primer pairs developed, 3264 pairs showed successful amplification as demonstrated by *in-silico* PCR validation against the argan genome. Additionally, we assessed the cross-species transferability of these primer pairs, revealing a low transferability rate in two *Sapotaceae* species (*Pouteria glomerata* and *Vitellaria paradoxa*) based on their draft genome sequences. Overall, these findings hold significant potential for both theoretical and applied research on argan, providing valuable insights and resources for further exploration and utilization.

## Supplementary Material

baae118_Supp

## Data Availability

All the data that support the findings of this paper are available within the paper and its supplementary materials published online.
